# Gait Recognition and Walking Exercise Intensity Estimation

**DOI:** 10.3390/ijerph110403822

**Published:** 2014-04-08

**Authors:** Bor-Shing Lin, Yu-Ting Liu, Chu Yu, Gene Eu Jan, Bo-Tang Hsiao

**Affiliations:** 1 Department of Computer Science and Information Engineering, National Taipei University, No. 151, University Road, Sanshia District, New Taipei City 23741, Taiwan; E-Mail: bslin@mail.ntpu.edu.tw; 2 Department of Electronic Engineering, National Ilan University, No. 1, Sec. 1, Shenlung Road, Yilan 260, Taiwan; E-Mail: chu@niu.edu.tw; 3 Department of Electrical Engineering, National Taipei University, No. 151, University Road, Sanshia District, New Taipei City 23741, Taiwan; E-Mail: gejan@mail.ntpu.edu.tw; 4 Graduate Institute of Biomedical Electronics and Bioinformatics, National Taiwan University, No. 1, Sec. 4, Roosevelt Road, Taipei 10617, Taiwan; E-Mail: geniustom@gmail.com

**Keywords:** gait recognition, exercise intensity, linear discriminant analysis (LDA), empirical mode decomposition (EMD)

## Abstract

Cardiovascular patients consult doctors for advice regarding regular exercise, whereas obese patients must self-manage their weight. Because a system for permanently monitoring and tracking patients’ exercise intensities and workouts is necessary, a system for recognizing gait and estimating walking exercise intensity was proposed. For gait recognition analysis, *αβ* filters were used to improve the recognition of athletic attitude. Furthermore, empirical mode decomposition (EMD) was used to filter the noise of patients’ attitude to acquire the Fourier transform energy spectrum. Linear discriminant analysis was then applied to this energy spectrum for training and recognition. When the gait or motion was recognized, the walking exercise intensity was estimated. In addition, this study addressed the correlation between inertia and exercise intensity by using the residual function of the EMD and quadratic approximation to filter the effect of the baseline drift integral of the acceleration sensor. The increase in the determination coefficient of the regression equation from 0.55 to 0.81 proved that the accuracy of the method for estimating walking exercise intensity proposed by Kurihara was improved in this study.

## 1. Introduction

Previous studies have shown that obesity leads to various medical conditions and diseases such as high cholesterol, coronary heart disease, cerebrovascular disease, hypertension, and diabetes [1]. To improve the health of patients with cardiovascular disease, physicians must continually monitor the adequacy of these patients’ exercise intensity and frequency. Physicians must evaluate the amount of exercise patients with cardiovascular disease require. Because performing regular exercise prevents obesity, identifying the appropriate exercise intensity required for optimizing health benefits is crucial. 

Metabolic measurement and targeted heart rate measurement are common methods used for evaluating exercise intensity levels. Metabolic measurement is the most accurate method; however, the equipment is expensive and lacks mobility. Thus, implementing targeted heart rate measurement seems more feasible than metabolic measurement. However, medicines used for treating cardiovascular diseases can affect heart rates. When patients take these medicines, their heart rates during exercise might not reflect the actual exercise intensity level. 

Because of the problems associated with using metabolic and targeted heart rate measurement, the metabolic equivalent (MET) was proposed to measure a person’s exercise intensity [2]. In 1990, Jette *et al*. defined one MET as the amount of oxygen consumed in 1 min while sitting at rest, which is equivalent to 3.5 mL of oxygen per kilogram of body weight, [3]. Currently, the most cited paper providing complete MET information is the *Physical Activity Guide*, which was published by the University of South Carolina [4,5]. In 2000, a new version of the physical guidelines produced by the American College of Sports Medicine (ACSM) defined various activities supplemented with corresponding exercise intensities [6]. Because the exercise intensity table developed by the University of South Carolina [4,5] is highly accurate, various researchers have begun to investigate how body movements and body inertia affect exercise intensity. Research results have indicated that an accelerometer can be adopted to identify body movements and calculate the work executed through body inertia [7,8,9,10,11,12]. Once classified, the intensity of a certain exercise item can be determined by consulting the MET table [5]. In a study conducted in 2012 [13], Kurihara attached accelerometers to participants’ waists to evaluate walking exercise intensity. He suggested a unique form of inertia, the work exerted that correlates to exercise intensity changes. When a person is walking, the exercise intensity is approximately the sum of the work exerted through body movement and the resting metabolic rate (RMR). By analyzing how a person walks at different velocities, a regression formula can be deduced to calculate the walking exercise intensity. 

Because the raw data acquired from accelerometers contain random noise and errors, Kurihara’s regression formula might be slightly inaccurate. The purpose of this study was to solve this problem by reexamining and verifying the accuracy of Kurihara’s conclusions. The experiments in this study were conducted using experimental conditions identical to those of Kurihara. The difference between the results obtained when applying the proposed improvements and those obtained when the improvements were not applied is discussed, and the results are compared with experimental data provided in ACSM literature [6].

## 2. System Architecture and Design

**Figure 1:**
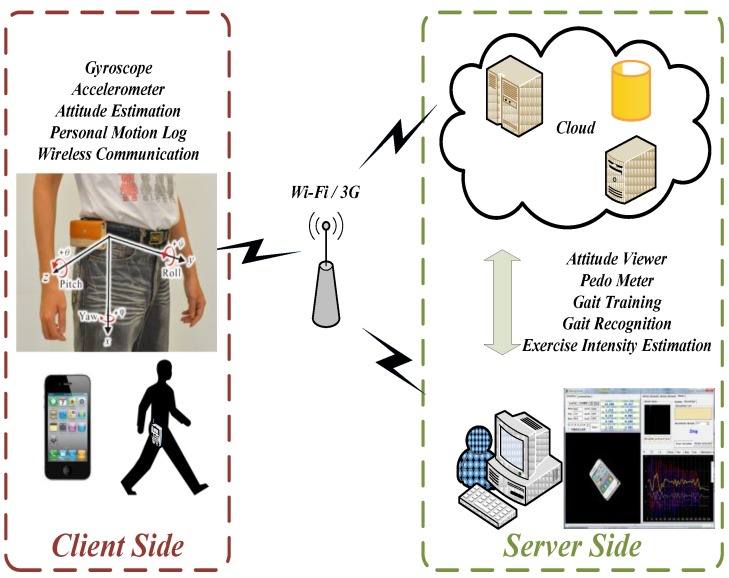
Overall system architecture.

Smartphones are currently integrated with accelerometers, gyroscopes, inertial navigation components, 3G communication, Wi-Fi, and Bluetooth; therefore, they can be used as portable devices for evaluating exercise and calculating calorie consumption. The proposed system architecture was divided into two parts, a client side and a server side (Figure 1).

The client-side system architecture is a portable device that collects data from an accelerometer and a gyroscope, and an attitude estimation algorithm was developed to calculate user attitude. The collected information is then sent to the server side through a wireless network for analysis.

A software interface was developed on the server side so that the users’ attitude can be displayed in 3D graphics and the gait recognition process can simultaneously be implemented. When the motion is recognized as a walking posture, the number of steps and walking exercise intensity are calculated. 

**Figure 2:**
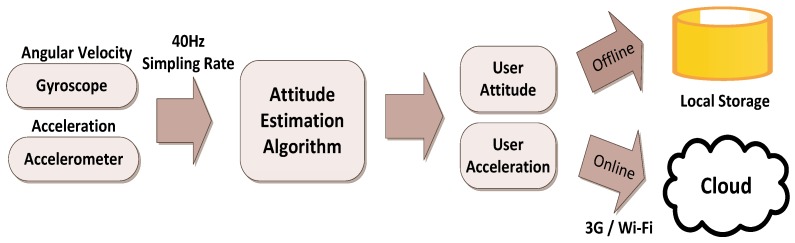
The flowchart of client-side operations.

The client side is a portable device equipped with a gyroscope, an accelerometer, and a Wi-Fi or 3G Internet interface. The device is attached to the waist, and a gait recognition algorithm installed on the device calculates the user’s gait. When the device is online, the calculations are transmitted to the server side through the Wi-Fi or 3G Internet interface in real time for subsequent analysis and verification. When the device is offline, the data are temporarily saved on a client side. When the connection is restored, all of the saved data are sent to the server. Figure 2 shows the client-side operational procedures.

The client device was implemented using the iPhone 4 because it is the first smartphone to be equipped with an accelerometer and a gyroscope and to provide an application programming interface (API) for the Core Motion Framework [14], thus facilitating the calculation of attitude. The accelerometer and gyroscope of the iPhone 4 are respectively the STM33DH (±1.7 g; STMicro) and AGD12022 (±250°/s; STMicro). The program was implemented using the Core Motion API to acquire raw data from the accelerometer and the gyroscope. In a previous study [15], the sampling rate was approximately 30 Hz; therefore, the sampling rate was defined as 40 Hz in this system. Apple Mac Air and XCode 5.0 were used to develop the program, and the operating system of the iPhone was iOS 5.0.

**Figure 3:**
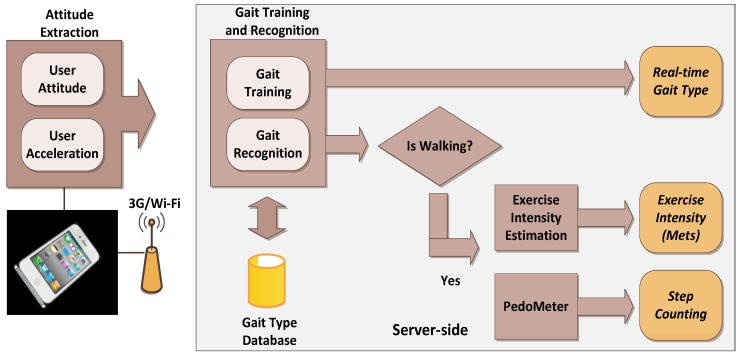
The flowchart of server-side operations.

Figure 3 shows the server-side operation procedures. When receiving data from a client, the server first graphically depicts the current attitude of the user by using OpenGL, and gait training or recognition is then performed using attitude angle and acceleration data. The data are stored in a gait type in a Microsoft Access database and can be dynamically updated and edited using a novel interface.

## 3. Methodology and Principles of Analysis

The smartphone initially calculates user attitude according to the sensor data, and then transmits the extracted data to a PC through a Wi-Fi or 3G network to execute gait training and recognition. When the system detects that the user is walking, the number of steps is calculated and the walking frequency results are displayed.

### 3.1.Attitude Recognition

#### 3.1.1. Attitude Representation

This study used a combination of the inertia component system (accelerometer and gyroscope) as the basis for attitude recognition. An inertial system was used to express attitude according to coordinate transformation. Euler angles were used to express the degree of postural change. Euler angle notation consists of the pitch, yaw, and roll angle formed in three directions; hence, the Euler angle is referred to as the attitude angle. In this study, smartphones were attached to patients’ waists to detect their gaits. Because a triaxial gyroscope can detect only instantaneous angular velocity, instantaneous attitude angles cannot be detected in the space. The accelerometer detected instantaneous acceleration even in the presence of gravity, *g*. when the gravitational acceleration *g* relative to the regulation of the three axial components can be extracted, the attitude angles in the space can be identified.

#### 3.1.2. Introduction to the *αβ* Filter

**Figure 4:**
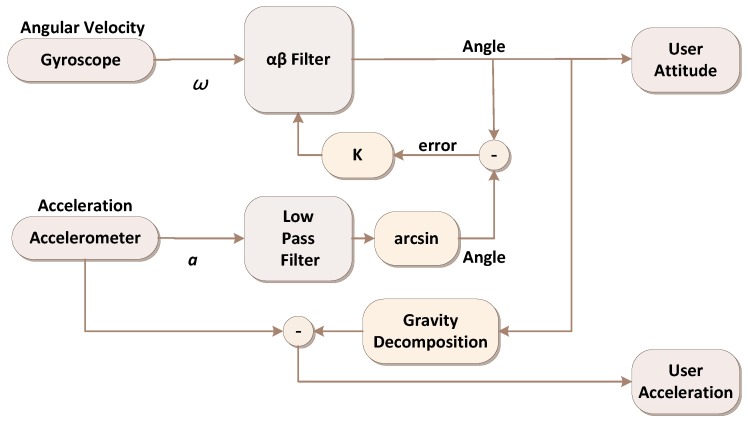
The flowchart of the gait recognition algorithm.

Previous studies have used *αβ* filters to calculate rapid changes in signals with a small phase delay [16]. Hence, an *αβ* filter was used to enable the accelerometers and the gyroscope to compensate for each other. The attitude angle detection algorithm involves the following steps: The accelerometer data pass through a low-pass filter (50 Hz), and an arcsine function is then used to calculate the attitude angles [17]. The computed attitude angles from the accelerometers and the attitude angles from the *αβ* filter compensate for each other, enabling an error to be obtained and then adjusted according to the gain *K*. Finally, the *αβ* filter calculates the user’s attitude angles in real space by using error convergence [18]. Figure 4 illustrates the procedures executed in the gait recognition algorithm adopted in this study. The three axial weights of acceleration values calculated corresponding to the attitude angles are reserved accelerations, except gravity.

The useful accelerations are detached from the unrelated gravity *g*; this process is known as gravity decomposition. Thus, the user’s attitude in space is obtained (pitch and roll), and the acceleration excluding the influence of gravitational acceleration (in X, Y, and Z directions) is shown.

### 3.2. Gait Training and Recognition

#### 3.2.1. Preprocessing the Gait Vector

**Figure 5:**
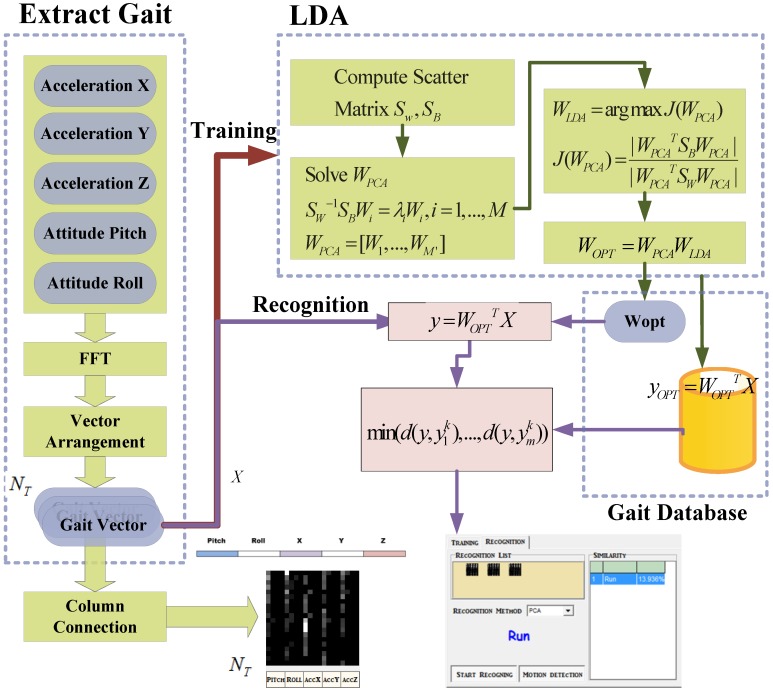
The flowchart of gait training and recognition algorithm.

After the user’s attitude is determined, the subsequent step is to recognize the user’s gait. Figure 5 shows the entire gait detection algorithm. First, the attitude obtained in the previous section, which consists of the pitch, roll, acceleration X, acceleration Y, and acceleration Z values, can be combined into a vector. After being processed using linear discriminant analysis (LDA), the optimal classification of projection matrix *W_opt_* and the vector classification can be obtained, and the training results are stored in the database. Hence, the attitude vector projected by *W_opt_* and the samples of the various categories stored in database are calculated together to obtain the Euclidean distance. When the Euclidean distance is minimal, the attitude is recognized as a gait.

**Figure 6:**
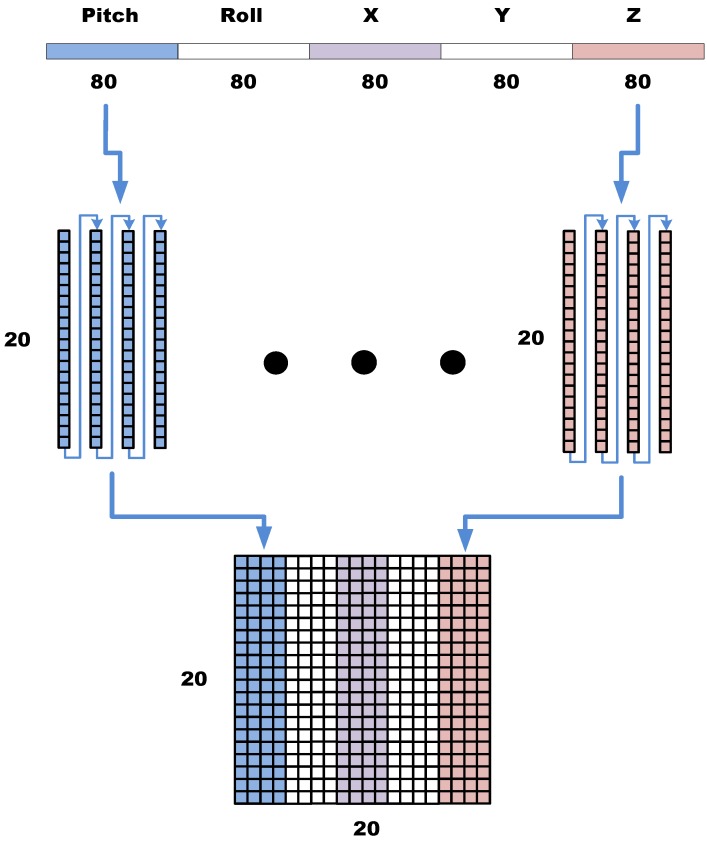
Representation of the gait vector transferring to an image.

To capture the gait, we conducted experiments to determine the variations in attitude and acceleration in the space. When an object was located on the horizontal rotation (yaw), gravity exerted no effect on the object and the accelerometer could not provide the correct compensation for horizontal displacement. Furthermore, horizontal rotations were observed when the user turned or changed direction. Our experiments indicated that the extracted yaw information was not useful in gait recognition; therefore, the yaw data were ignored. In this study, the sampling rate of the sensors was 40 Hz, a gait feature vector defined as 1.5 times the longest step period, 2.7 s [19], was used as a window size, and 160 samples were used. The data had to be transformed into frequency spectra before recognition or training could be applied, because the samples comprised time domain information. When several gait feature vectors were extracted from any point of a continuous time domain datum, the feature vectors did not exhibit similar features. However, after the data were transformed into the frequency domain, the feature vectors were similar regardless of the time at which the 160 samples were extracted. Thus, transforming data in time domain into the frequency domain improved the outcome of the subsequent training or recognition procedure. In this study, the fast Fourier transform (FFT) was used to extract spectral energy. Because the information was converted to symmetrical spectra [20], we allowed the 160 coefficients obtained from the FFT to be reduced to the former 80 coefficients for subsequent processing. Using this method reduced the complexity of subsequent calculations and enabled the gait vector *I_m _*= {*x_1_*, *x*_2_, … , *x*_400_} to be constructed by connecting the pitch, roll, acceleration X, acceleration Y, and acceleration Z in a series. In the next step, the gait vector must be connected in a series of columns and transformed into the image shown in Figure 6. The serial representation converts the spectra into grayscale images, thereby intensifying the differences among various gaits.

**Figure 7:**
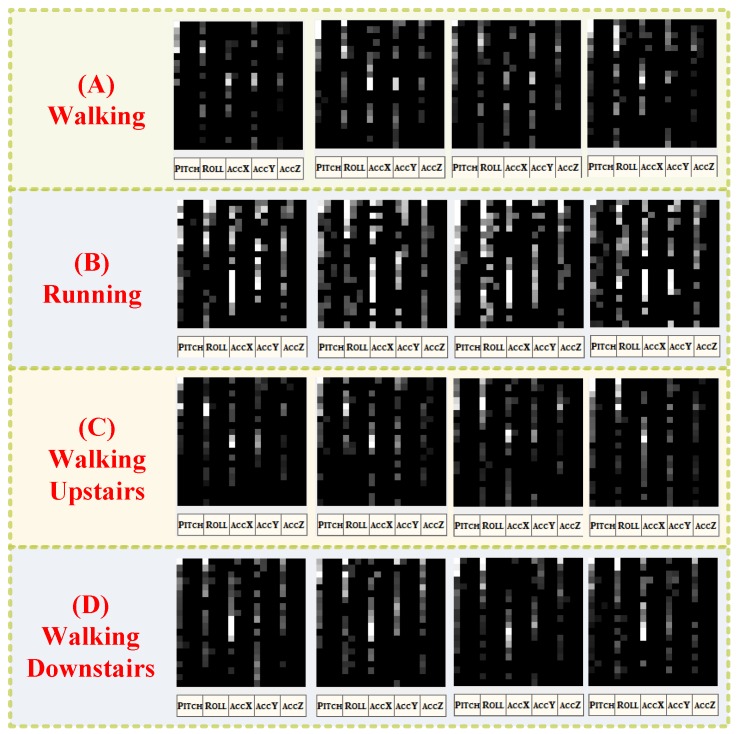
Spectral images of gait in walking, running, walking upstairs, and walking downstairs.

Figure 7 shows the gait spectra of a participant walking, running, walking upstairs, and walking downstairs. For each gait, 160 points were randomly sampled, and the first 80 coefficients calculated according to the FFT were then extracted to form a spectral image. Consequently, similar gaits yielded similar grayscale distributions, and different gaits were clearly differentiated. Feature extraction and recognition were performed after the gait vectors were obtained.

#### 3.2.2. Decision Methods Used for Gait Recognition

In this study, the recognition method was based on the Euclidean distance. When the tested characteristic parameters were compared with all of the trained characteristic parameters, the category that exhibited the minimal Euclidean distance was recognized as the gait category.

### 3.3. Number of Steps and Period of Strides

**Figure 8:**
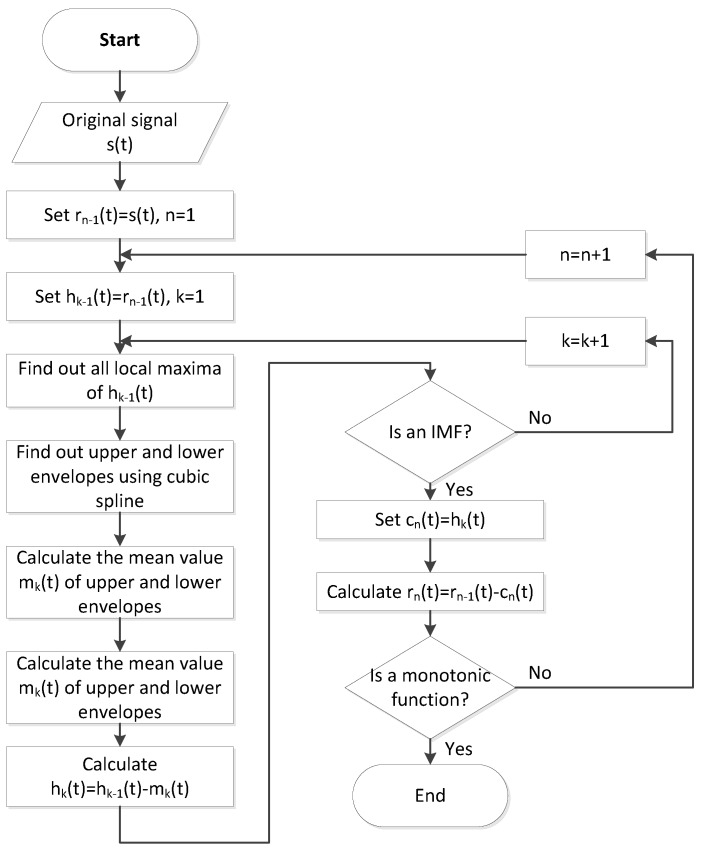
The flowchart of the EMD algorithm.

When walking is detected using gait recognition, the steps are counted to assist users in understanding their walking steps and stride frequency. In previous studies in which accelerometers were used for counting steps, the approaches mostly involved the time required to cross a zero point or threshold. However, irregularities in movement and other noise increase or reduce the times at which the zero point is crossed, thus causing erroneous judgments. In Figure 8, the curve of the original acceleration signal frequently crosses the zero axis, and when the threshold is used to filter noise, the results are extremely unreliable because the threshold method is not suitable for all users. Moreover, the walking postures and stride frequencies of the participants differed. This paper presents a method in which empirical model decomposition (EMD) [21] is used to calculate the number of steps and the orthogonal index (OI) test is used to enhance the validity of the EMD results. EMD entails adaptively and locally decomposing any nonstationary time series in a sum of intrinsic mode functions (IMFs) that represent zero-mean amplitudes and frequency-modulated components. An IMF can be represented as a series of sets of local orthogonal basis functions in which amplitude and frequency may vary over time. An IMF has two features: only one extreme exists between two subsequent zero crossings and the mean value is zero. EMD uses the sifting process to decompose the signal *s*(*t*) into IMFs as follows: 

Step 1: Identify all local maxima and local minima of *s*(*t*) and then use cubic splines in interpolating the local maxima and local minima to form upper and lower envelopes, respectively.

Step 2: Calculate the mean envelope according to the mean values of the upper and lower envelopes.

Step 3: Subtract the mean envelope from the original signal *s*(*t*) to obtain the first weight *h_1_*(t); that is *h_1_*(*t*) = *s*(*t*) − *m_1_*(*t*).

Step 4: Determine whether *h_1_*(*t*) fits the condition of the IMF. If it does not fit, then the process returns to Step 1 and *h_1_*(*t*) becomes the original signal the second time the fit is determined; that is, *h_2_*(*t*) = *h_1_*(*t*) − *m_2_*(*t*). Repeat *k* times for *h_k_*(*t*) = *h_k-1_*(*t*) − *m_k_*(*t*) until *h_k_*(*t*) fits the condition of the IMF. Eventually, the first IMF *c_1_*(*t*) can be obtained; that is, *c_1_*(*t*) = *h_k_*(*t*) .

Step 5: Subtract *c*_1_(*t*) from the original signal *s*(*t*) to obtain a residual function *r_1_*(*t*) ; that is, *r_1_*(*t*) = *s*(*t*)-*c_1_*(*t*) .

Step 6: Let *r_1_*(*t*) become the new material and reexecute Steps 1 through 5 to obtain the new residual function *r_2_*(*t*) . Repeat *n* times for *r_n_*(*t*) = *r_n_*_−1_(*t*) − *c_n_*(*t*) until the residual function *r_n_*(*t*) becomes a monotonic function. When *r_n_*(*t*) is a monotonic function, an IMF cannot be decomposed further and the EMD process is complete. 

A flowchart of the EMD algorithm is shown in Figure 8. EMD involves decomposing a data set *s*(*t*) into IMFs *c_n_*(*t*) and a mean trend such that the signal can be represented as:

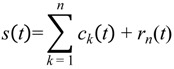
(1)

One effective method to verify that IMFs were decomposed through EMD is to determine whether any two IMFs are orthogonal. The goal of the OI test is to determine the orthogonality of IMFs created through EMD. The OI is defined as:

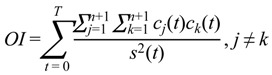
(2)

**Figure 9:**
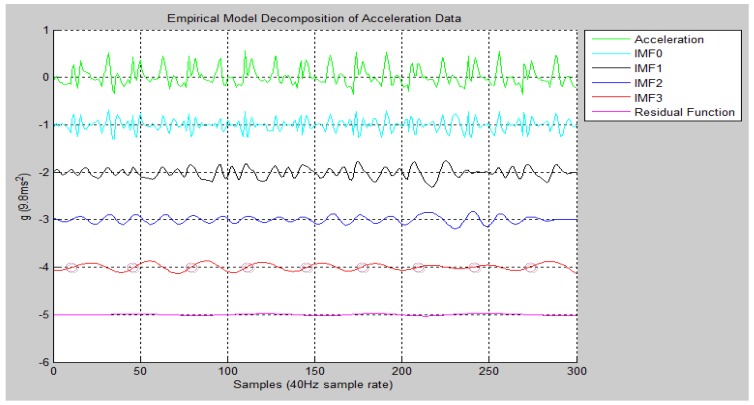
EMD results of acceleration data for walking 9 steps.

An OI value of approximately zero indicates that the orthogonality of IMFs is favorable; in other words, the IMFs are reliable. For example, Figure 9 shows acceleration data and the EMD for walking nine steps. When EMD was used to decompose the original acceleration of signals, IMF0 to IMF2 were noise-like signals that exhibited irregular zero crossing distributions. However, IMF3 appeared highly regular and its number of zero crossings was completely consistent with the number of steps walked. By using this method, we discovered that, after applying EMD, walking step information could be obtained based on the zero crossings produced by IMF3. 

**Figure 10:**
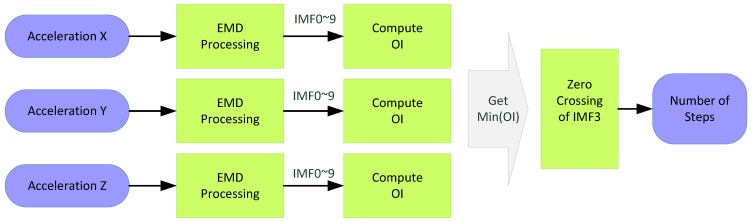
Flow diagram of counting the number of walking steps.

Because the data of all triaxial directions of an accelerometer can change while a user is walking, the signals of the three axial accelerations individually pass through the EMD to obtain their OIs. The axial direction that exhibited the minimal OI value was used as the reference for counting the steps walked and the zero crossings of IMF3. Finally, the number of steps walked was obtained. A flow diagram of the procedures performed when counting the number of steps walked is shown in Figure 10.

### 3.4. Estimation of Exercise Intensity

When the system detects an attitude that indicates that the user is walking and counts the steps, the exercise intensity calculation begins. The error in the inertial element must be minimized before calculating motion strength. In a previous study [22], a signal inaccuracy equation indicated the cause of error. The equation is expressed as Formula (3):

*f_a_* = *f* + *b_a_* + *S_1_f* + *S_2_f^2^* + *Nf* + *δg* + *ε* (3)

where *f_a_* is the measured acceleration; *f* is the true specific acceleration; *b_a_* is the accelerometer instrument bias; *S_1_* and *S_2_* are the linear and nonlinear scale factor matrices, respectively; *N* is the nonorthogonality matrix; *δg* is the deviation from theoretical gravity; and *ε* is the accelerometer noise.

According to Formula (3), the inaccuracy equation of the accelerometer is a quadratic equation. In our proposed system, *δg* was removed in preprocessing, *b_a_* was seemed as a baseline drifted dc component, and *ε* is the random noise that should be processed separately. Hence, let *Q* equal *S_2_f^2^* + (*S_1_ + 1 + N*) *f + b_a_*. Formula (3) becomes a standard quadratic equation with random noise. This new equation can be rearranged as Formula (4):

*f_a_* = *Q* + *ε*, *Q = S*_2_*f*^2^ + (*S*_1_ + 1 + *N*) *f* + *b_a_* (4)

**Figure 11:**
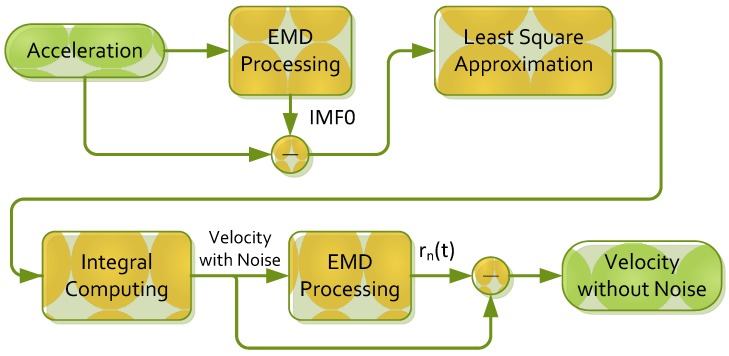
The flowchart of filtering acceleration noise.

The EMD algorithm was applied to remove the effect of random noise *ε*. In EMD processing, IMF0 exhibited the highest frequency component among all IMFs; thus, most random noise was located in IMF0. Therefore, the random noise was assumed to be filtered when the IMF0 was subtracted from the original signal. To reduce the effect of errors, in addition to random noise *ε*, a quadratic function *Q* must be processed. In this study, a least-square approximation was used to process a quadratic approximation for acceleration data (in X, Y, and Z directions); in other words, a quadratic function exhibiting a minimal sum of distances between all points was identified. After being subjected to the least-square approximation, the acceleration was integrated to obtain a velocity and a constant term, which was a dc component. Finally, the velocity was subtracted from a residual function *r_n_*(*t*) to obtain a reliable velocity value. A flowchart of acceleration noise filtering is shown in Figure 11.

After the real walking speed was obtained, the physical inertia formula proposed by Kurihara [13] was used to calculate the exercise intensity:

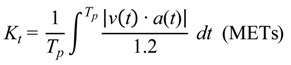
(5)
where *K_t_* is the work done by inertia while walking; *T_p_* is the traveling time; and *v*(*t*) and *a*(*t*) are respectively the instantaneous velocity and the instantaneous acceleration. The system calculates the work done by inertia while walking by using Formula (5).

## 4. Experimental Results and Discussion

### 4.1. Algorithm Verification

Algorithm verification involved verifying users’ acceleration, attitude, and exercise intensity. In the tests, 10 people (five men and five women) participated and 100 walking data records were collected (10 records for each of the 10 participants). The participants walked along a 50-m straight line and maintained a speed of approximately 1.56 m/s in every test. The effects of age, gender, height, weight, and leg length on the results were analyzed, and basic information regarding the participants is listed in Table 1. 

**Table 1:** The age, gender, height, weight, and leg length of the 10 participants.

Gender	Male	Female
Subjects	5	5
Age	30 ± 4	26.5 ± 3.5
Height	172 ± 9 cm	155 ± 5 cm
Body Weight	75 ± 10 Kg	47.5 ± 6.5 Kg
Leg Length	101 ± 9 cm	87.5 ± 2.5 cm

The Leica Disto™ D3ABT [23] (Leica Geosystems, Heerbrugg, Switzerland) was used to validate the proposed system because it features a sensing range from 5 cm to 100 m and a measurement error within ±1 mm. This distance measuring equipment (DME) is equipped with a Bluetooth interface that can be adopted to transfer the data on the distance between the equipment and the target to a PC. A program installed on the PC was used to synchronously collect, store, and analyze data from the DME and iPhone 4. According to Newton’s laws of motion, velocity is the first derivative of distance over time, and acceleration is the second derivative of distance over time. Thus, the first and second derivatives of the distance measured using the DME were calculated to determine the velocity and acceleration, which were then compared with the results generated by the proposed algorithm. 

**Figure 12:**
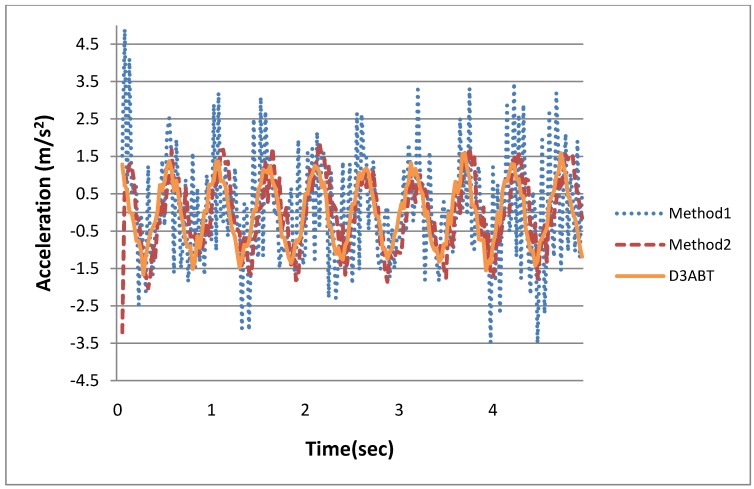
The comparison of walking accelerations of D3ABT, Method1, and Method2.

In this experiment, the exact walking distance had to be measured for the subsequent differential process. Therefore, we set up a tripod equipped with a rangefinder at the starting point of a walkway, and the laser rangefinder was aimed at a user’s waist. To describe and discuss the results, we denote the results obtained when using the laser rangefinder as D3ABT, the results of reduplicating Kurihara’s algorithm as Method1, and the results of the proposed algorithm as Method2. After the second derivative was measured and processed, the walking accelerations of D3ABT, Method1, and Method2 were obtained. A comparison is shown in Figure 12, which indicates that Method1 exhibited more noise and drift than did Method2 and D3ABT. Method2, which was similar to D3ABT, was obtained by using IMF0 and least-square approximation to remove random noise and to reduce the effect of the quadratic inaccuracy equation.

**Figure 13:**
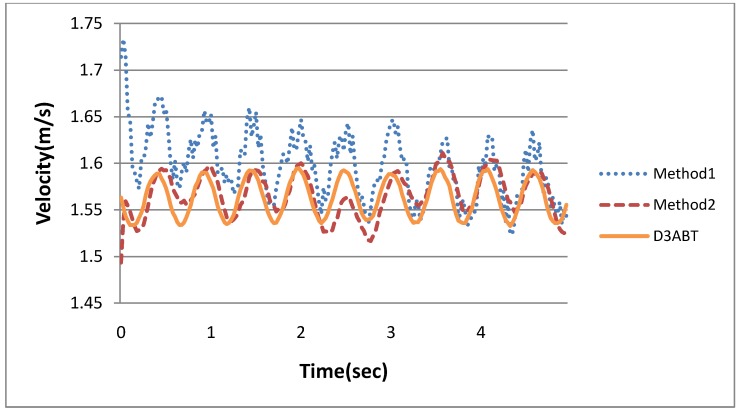
The comparison of walking velocities of D3ABT, Method1, and Method2.

Subsequently, the accelerations shown in Figure 12 were converted into velocities. The results are shown in Figure 13, which indicates that Method1 exhibited greater error than did D3ABT. Method2 also exhibited error, but the error of Method2 was much smaller than that of Method1. Method1 exhibited error because the acceleration noise was not filtered sufficiently, causing critical drift and amplitude amplification after integration. These errors exerted adverse effects on subsequent calculations of exercise intensity. To resolve this problem, the algorithm illustrated in Figure 11 was proposed. The integrated data were subtracted from the residual function to reduce the effect of baseline drift.

**Figure 14:**
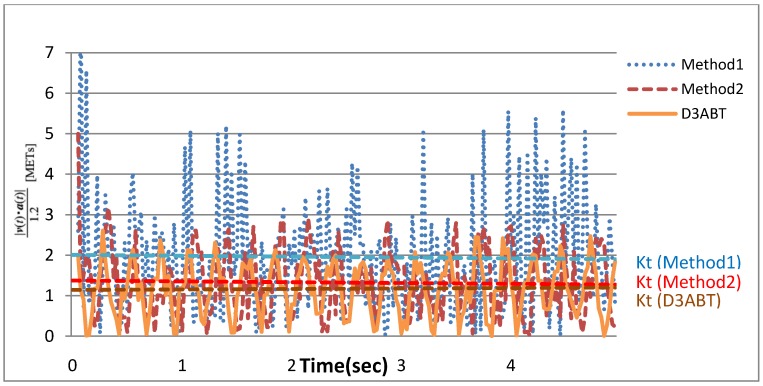
The comparison of work done by inertia *K_t_* of D3ABT, Method1, and Method2.

Kurihara defined work done by inertia *K_t_* as a sum of |*v*(*t*) ∙ *a*(*t*)| during a unit of time (*i.e.*, Formula (5)). In this experiment, |*v*(*t*) ∙ *a*(*t*)| and *K_t_* were obtained by calculating the accelerations and velocities of D3ABT, Method1, and Method2. A comparison of *K_t_* is shown in Figure 14, which indicates that *K_t_* of Method1 was much greater those of Method1 and D3ABT. Method1 exhibited error because of acceleration error, which amplified the velocity error several times after integration and increased the error in |*v*(*t*) ∙ *a*(*t*)| that was required to calculate exercise intensity. Therefore, when exercise or walking intensifies or becomes faster, the error in *K_t_* of Method1 is expected to be much greater than those of Method1 and D3ABT.

### 4.2. Experiments on the Number and Frequency of Steps

In this experiment, 10 participants (five men and five women) participated in the tests; the detailed information is listed in Table 1. Every participant walked 10 times, ran two times, walked upstairs two times, and walked downstairs two times.

Every participant walked 10 times along a 50-m-long covered corridor. The first six times, the participants walked at a speed that they perceived to be normal. The final 4 times, the participants walked at one speed that they perceived to be fast, one speed that they perceived to be very fast, one speed that they perceived to be slow, and one speed that they perceived to be very slow. The participants counted the number of steps and then reported this number to the experienced investigator when they reached the end of the walking trial. Subsequently, the investigator recorded the time and the number of steps, and acceleration data (in the X, Y, and Z directions) and gyroscope data (pitch, yaw, and roll) from the iPhone 4 were stored on the server side. The walking speed was varied to obtain a mean distribution of speeds for calculating the exercise intensity and to improve the reliability of the regression equation of exercise intensity.

In addition, every participant ran two times along a 50-m-long covered corridor at a speed that they perceived to be normal. The subsequent procedures were identical to those of the walking tests. Finally, the experienced investigator recorded the time and number of steps, and acceleration data (in the X, Y, and Z directions) and gyroscope data (pitch, yaw, and roll) from the iPhone 4 were stored on the server side.

Every participant walked upstairs two times and downstairs two times. The testing environment encompassed the fifth to seventh floors of a building. One platform was located between every two floors, and 11 stairs were located between a platform and a floor; thus, the total number of stairs was 44. Subsequently, the participants walked upstairs and downstairs at a speed that they perceived to be normal. The subsequent procedures were identical to those of the walking tests. Finally, the investigator recorded the time and the number of steps, and acceleration data (in the X, Y, and Z directions) and gyroscope data (pitch, yaw, and roll) from the iPhone 4 were stored on the server side.

In this experiment, 100 walking data records were collected (10 records for each of the 10 participants). Subsequently, the number of steps that the participants counted was compared with the number of steps calculated by the system to determine the accuracy of the system. Finally, the causes of errors were identified, and methods for improvement were then suggested. Table 2 lists the step detection accuracies of the 100 walking data records.

Detection accuracy was calculated using Formula (6).

(6) 100% − (steps counted by the participants − steps detected by the system)/ (steps counted by the participants) %

Referring to Formula (6), the results indicated that gender did not influence step detection accuracy. The accuracy ranged between 93% and 97%, and the average accuracy was 95%. By analyzing the causes of detection errors, we discovered that the errors in the steps resulted from body swinging before and after the experiments or from miscalculations by the participants. 

**Table 2:** Step detection accuracy.

	Gender	Male					Female				
	Subject	Subject1	Subject2	Subject3	Subject4	Subject5	Subject6	Subject7	Subject8	Subject9	Subject10
Walking Speed	Normal	98.75%	91.67%	96.67%	91.89%	96.05%	96.77%	96.77%	100.0%	96.00%	96.00%
	Normal	96.25%	97.22%	100.0%	92.11%	93.75%	98.92%	98.92%	96.20%	95.89%	90.91%
	Normal	94.29%	95.71%	90.00%	94.81%	95.31%	94.68%	94.68%	95.95%	96.00%	86.84%
	Normal	96.30%	98.41%	93.44%	96.05%	100.0%	94.57%	94.57%	97.37%	100.0%	91.89%
	Normal	97.22%	92.06%	93.44%	97.33%	92.19%	93.26%	93.26%	93.42%	97.30%	89.04%
	Normal	98.75%	89.29%	96.72%	94.81%	90.63%	97.75%	97.75%	97.40%	94.03%	96.00%
	Fast	92.86%	91.07%	100.0%	96.05%	91.38%	90.00%	90.00%	95.31%	95.65%	97.06%
	Very Fast	98.86%	93.02%	98.39%	97.33%	85.19%	88.24%	88.24%	88.33%	83.33%	93.33%
	Slow	95.12%	98.46%	96.72%	100.0%	97.10%	95.79%	95.79%	91.14%	93.83%	92.11%
	Very Slow	97.56%	100.0%	98.36%	95.65%	98.61%	98.92%	98.92%	98.77%	78.02%	98.82%
Average Accuracy		96.60%	94.69%	96.37%	95.60%	94.02%	94.89%	94.89%	95.39%	93.01%	93.20%
Gender Accuracy		95.46%					94.28%				
Overall Accuracy		94.87%									
				>95%		90~95%		<90%			

### 4.3. Gait Recognition Experiments

**Figure 15:**
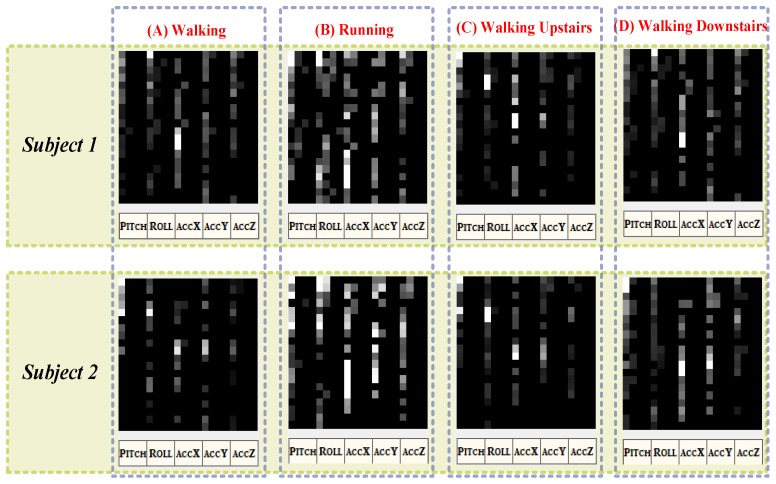
The spectral energy diagrams of walking, running, walking upstairs and walking downstairs from subject 1 and subject 2.

Spectral energy diagrams of pitch, roll, acceleration X, acceleration Y, and acceleration Z were produced while the participants (the detailed information is listed in Table 1), ran, walked, walked upstairs, and walked downstairs. Figure 15 shows that the distributions of the spectral energy diagrams of two participants’ gaits differ.

Table 3 shows the gait-recognition accuracy analysis of the participants. Each participant was required to walk, run, walk upstairs, and walk downstairs. Each category comprised 60 samples, and 240 samples were collected from each participant; thus, a total of 2,400 samples were collected from the participants. Table 4 shows that, when the ratio of the training data to all collected data was over 30%, the recognition rate remained higher than 90%. When the percentage was under 20%, the recognition rate decreased because of the number of samples was insufficient (under 60 samples × 20% = 12 samples). Thus, when the ratio of training data to all collected data was increased, the recognition rate increased.

**Table 3:** The recognition results of walking, running, walking upstairs and walking downstairs from ten subjects.

Subjects	Percentage of Training Data (%)								
	10%	20%	30%	40%	50%	60%	70%	80%	90%
Subject 1	78.02%	88.65%	95.60%	97.10%	97.80%	98.50%	99.50%	100.00%	100.00%
Subject 2	75.07%	85.93%	92.98%	94.48%	94.30%	96.07%	96.45%	96.95%	98.24%
Subject 3	74.91%	84.67%	93.57%	95.07%	96.67%	97.93%	96.30%	96.80%	98.47%
Subject 4	75.03%	86.05%	92.03%	93.53%	96.95%	98.02%	95.85%	96.35%	98.89%
Subject 5	75.75%	87.06%	92.29%	93.79%	93.42%	97.09%	97.30%	97.80%	96.96%
Subject 6	75.24%	85.90%	91.58%	93.08%	94.06%	96.94%	94.96%	95.46%	100.00%
Subject 7	75.89%	85.56%	94.50%	96.00%	95.31%	94.38%	96.91%	97.41%	99.53%
Subject 8	76.66%	87.65%	93.53%	95.03%	94.75%	95.11%	96.56%	97.06%	99.03%
Subject 9	74.93%	85.39%	90.83%	92.33%	95.00%	96.54%	97.87%	98.37%	99.88%
Subject 10	76.51%	88.03%	93.09%	94.59%	93.53%	94.40%	96.66%	97.16%	99.46%
Average Recognition Rate(%)	75.80%	86.49%	93.00%	94.50%	95.18%	96.50%	96.84%	97.34%	99.05%
Worst Recognition Rate(%)	74.91%	84.67%	90.83%	92.33%	93.42%	94.38%	94.96%	95.46%	96.96%
Best Recognition Rate(%)	78.02%	88.65%	95.60%	97.10%	97.80%	98.50%	99.50%	100.00%	100.00%

### 4.4. Exercise Intensity Experiments

To validate the calculated exercise intensity, the gait information obtained from each person was analyzed. The walking speed (*V_w_*), single-point velocity (*v*), single-point acceleration (*a*), work done by inertia (*K_t_*), and walking exercise intensity (*K_walk_*) were calculated using 100 walking samples collected from the 10 participants and various algorithms to analyze the correlation coefficients. 

**Figure 16:**
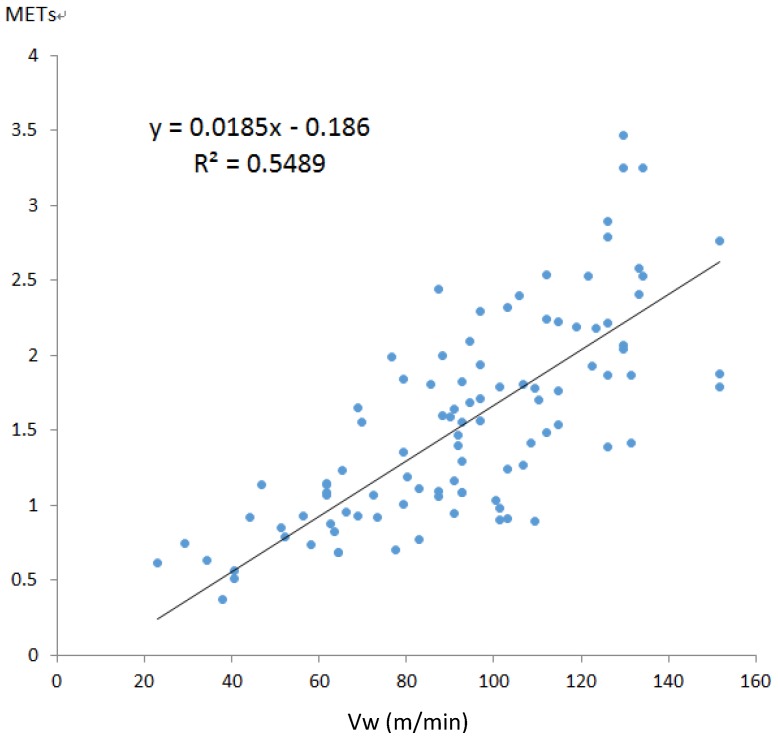
Results of Kurihara’s method reduplicated in this study.

**Figure 17:**
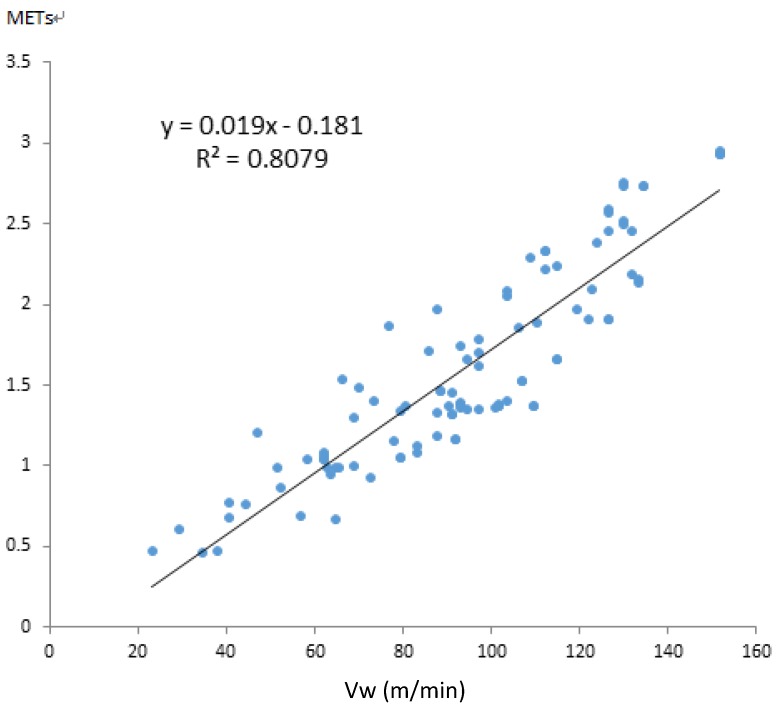
Results of the improved velocity calculation algorithm developed in this study.

To verify that the proposed algorithm is accurate, the calculation results were compared with those obtained using Kurihara’s algorithm. The work done by inertia in the 100 data records of the 10 participants was calculated. Figure 16 illustrates the results obtained using Kurihara’s algorithm, and Figure 17 illustrates the results obtained using the improved velocity calculation algorithm developed in this study. In the figures, the walking distance per minute, *V_w_* , is plotted along the x-axis, and the METs of the work done by inertia corresponding to various velocities are plotted along the y-axis.

According to the test results of Kurihara [13] and the results obtained by duplicating Kurihara’s method, the number of errors increased as the velocity increased. By contrast, Figure 17 shows that the deficiency was greatly improved using the method developed in this study. Investigating the cause revealed that, when using the accelerometer, increasing the velocity resulted in higher data fluctuations, thereby increasing the amount of noise and the error.

### 4.5. Discussion

In the preparation phase of this study, EMD was adopted to filter out the white noise of IMF0. Moreover, when calculating the velocity, the proposed method was used to filter out the second-order noise inherent in the accelerometer and the baseline drift through the residuals of EMD. Therefore, even when the walking velocity increased, the errors were not amplified.

The results of Kurihara and those obtained by duplicating Kurihara’s method exhibited coefficients of determination of 0.59 and 0.55, respectively. This difference was observed because Kurihara collected five data records on 16 participants, amounting to 80 data records; by contrast, 100 data records were collected in this study. Consequently, the increased number of data records slightly reduced the coefficients of determination to 0.55. The results plotted in Figure 17 were obtained using the new algorithm proposed in this study, and the coefficient of determination was enhanced to 0.81 because the accuracy of acceleration and velocity calculation was improved.

Kurihara’s regression equation is expressed as follows:

*K_t_* = 0.022*V_w_* ‒ 0.19 (7)

Kurihara’s regression equation was duplicated in this study as follows:

*K_t_* = 0.0185*V_w_* ‒ 0.186 (8)

The improved regression equation is expressed as follows:

*K_t_* = 0.019*V_w_* ‒ 0.181 (9)

*K_t_* is the work done by inertia, and *V_w_* is the walking velocity per minute. The first-degree term and constant term in this study were almost identical to those reported by Kurihara. The walking velocity per minute *V_w_* in *K_t_* = 0.019*V_w_* − 0.181 can be rewritten as follows:

*V_w_* = 52.6 *K_t_* + 9.5 (m/min) (10)

According to Waters [24], the relationship between *V_w_* and *K_walk_* is expressed as follows:

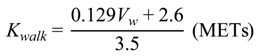
(11)

Substituting Formula (10) into Formula (11) yields the following equation:

*K_walk_* = 1.93*K_t_* + 1.09 (METs) (12)

According to Kurihara, Formula (12) indicates that the walking exercise intensity (*K_walk_*) is the sum of 1.93 times the work done by inertia and RMR (1.09 METs). The first-degree term indicates that walking involves the work done by inertia, the force of friction, and the work done by rotation, and that the total consumption is 1.93 times the work done by inertia (*K_t_*). In addition, the constant term indicates that *K_t_* equals to zero when a participant is still. Theoretically, the RMR of *K_walk_* when the participant is not moving is approximately 1 MET, and this value that corresponds with the experimental result (1.09 METs). 

According to the results of duplicating Kurihara’s method, the relationship between *K_walk_* and *K_t_* is expressed as follow:

*K_walk_ =* 1.99*K_t_ +* 1.12 (METs) (13)

The formula provided in the ACSM and University of Southern California literature and Formula (13), which was developed by duplicating Kurihara’s method, were compared with the proposed Formula (12), as shown in Table 4.

In Table 4, *V_w_* is the walking velocity (m/min), and *K_walk_* represents the walking exercise intensity determined according to the MET table proposed in the ACSM and University of Southern California literature. 

The variable *K_walk_* is the value of walking exercise intensity calculated by substituting *V_w_* into (13), and 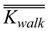 is the walking exercise intensity calculated by substituting *V_w_* into (12). The values |*Error*| and 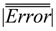 are the absolute values of *K_walk_* − *K_walk_* and 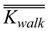 − *K_walk_* , respectively. Because the exercise intensity ranged from 0.9 to 15 [25], the average absolute error was defined as 100% × |Error| / (15 − 0.9). Table 4 indicates that the proposed algorithm increased the coefficients of determination of the regression equation from 0.55 to 0.81, and the regression equation exhibited an average error rate as low as 2.4%, which is superior to that obtained by duplicating the method of Kurihara (4%). These statistics verified that, compared with Kurihara’s method (13), the formula developed in this study, Formula (12), is more suitable for calculating the work done by inertia in the estimation of exercise intensity. 

**Table 4:** Comparison of accuracy of corresponding formulas in ACSM, reduplicated Y. Kurihara’s work, and our proposed system.

ACSM [6]		Reduplicated Y. Kurihara’s Work			The Proposed Sysem		
*V_w_* (m/min)	*K_walk_* (METs)	*K_walk_* (METs)	|*Error*| (METs)	*Error* percentage (%)	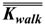 (METs)	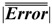 (METs)	*Error* percentage (%)
53.6	2	3	1	7.1%	2.6	0.6	4.3%
67	3	3.5	0.5	3.5%	3.1	0.1	0.7%
80.5	3.3	4	0.7	5.0%	3.6	0.3	2.1%
93.9	3.8	4.5	0.7	5.0%	4.1	0.3	2.1%
107.3	5	5	0	0.0%	4.6	0.4	2.8%
Mean			0.6	4%		0.4	2.4%
Standard Deviation			0.3	2.4%		0.2	1.2%

## 5. Conclusions

This study proposed a feasible personalized system for exercise intensity recording and gait recognition, which involved exercise posture capturing and exercise intensity estimation. In exercise posture capturing, an *αβ* filter was used to apply the triaxis angular velocity exported from the gyroscope to calibrate the accelerometer. Thus, real user motion acceleration excluding the effect of gravity was derived to estimate exercise intensity. Moreover, the direction information obtained from the gravity data of the accelerometer was used to calibrate the gyroscope, enabling the system to output precise motion posture and direction angle data used in the pretreatment of gait recognition. In gait recognition, four motion postures were used: walking, running, walking upstairs, and walking downstairs. The pitch, roll, acceleration X, acceleration Y, and acceleration Z information captured from the motion postures were subjected to FFT to form spectrograms. 

LDA training was used to process 30% of the images, and the remaining 70% were used for verification. The recognition accuracy rate was higher than 90%. To estimate exercise intensity, this study investigated the relationship between walking exercise intensity and the work done by inertia, analyzed the effects of the error equation of the accelerometer on the integration results, and proposed an algorithm integrating the quadratic approximation and residual functions extracted through EMD to filter the accelerometer noise that affects integration. The coefficient of determination of the regression equation increased from 0.55 to 0.81, thus proving that our proposed method improves the accuracy of walking exercise intensity estimation compared with the method of Kurihara.
